# High performance methylated DNA markers for detection of colon adenocarcinoma

**DOI:** 10.1186/s13148-021-01206-2

**Published:** 2021-12-13

**Authors:** Romy A. M. Klein Kranenbarg, Abdul Hussain Vali, Jan N. M. IJzermans, Thomas R. Pisanic, Tza-Huei Wang, Nilofer Azad, Saraswati Sukumar, Mary Jo Fackler

**Affiliations:** 1 Department of Oncology, Johns Hopkins University School of Medicine, Baltimore, MD USA; 2 Department of Medical Oncology, Erasmus MC Cancer Institute, University Medical Center Rotterdam, Rotterdam, The Netherlands; 3 Department of Surgery, Erasmus MC, University Medical Center Rotterdam, Rotterdam, The Netherlands; 4 Institute for NanoBioTechnology, Johns Hopkins University, Baltimore, MD USA; 5 Breast and Ovarian Cancer Program, Sidney Kimmel Comprehensive Cancer Center at Johns Hopkins, 1650 Orleans Street, CRB 1-Rm 144, Baltimore, MD 21231 USA

**Keywords:** Colon adenocarcinoma, Detection, Methylation, QM-MSP, cMethDNA, Liquid biopsy

## Abstract

**Background:**

Colon cancer (CC) is treatable if detected in its early stages. Improved CC detection assays that are highly sensitive, specific, and available at point of care are needed. In this study, we systematically selected and tested methylated markers that demonstrate high sensitivity and specificity for detection of CC in tissue and circulating cell-free DNA.

**Methods:**

Hierarchical analysis of 22 candidate CpG loci was conducted using The Cancer Genome Atlas (TCGA) COAD 450K HumanMethylation database. Methylation of 13 loci was analyzed using quantitative multiplex methylation-specific PCR (QM-MSP) in a training set of fresh frozen colon tissues (*N* = 53). Hypermethylated markers were identified that were highest in cancer and lowest in normal colon tissue using the 75th percentile in Mann–Whitney analyses and the receiver operating characteristic (ROC) statistic. The cumulative methylation status of the marker panel was assayed in an independent test set of fresh frozen colon tissues (*N* = 52) using conditions defined and locked in the training set. A minimal marker panel of 6 genes was defined based on ROC area under the curve (AUC). Plasma samples (*N* = 20 colorectal cancers, stage IV and *N* = 20 normal) were tested by cMethDNA assay to evaluate marker performance in liquid biopsy.

**Results:**

In the test set of samples, compared to normal tissue, a 6-gene panel showed 100% sensitivity and 90% specificity for detection of CC, and an AUC of 1.00 (95% CI 1.00, 1.00). In stage IV colorectal cancer plasma versus normal, an 8-gene panel showed 95% sensitivity, 100% specificity, and an AUC of 0.996 (95% CI 0.986, 1.00) while a 5-gene subset showed 100% sensitivity, 100% specificity, and an AUC of 1.00 (95% CI 1.00, 1.00), highly concordant with our observations in tissue.

**Conclusions:**

We identified high performance methylated DNA marker panels for detection of CC. This knowledge has set the stage for development and implementation of novel, automated, self-contained CC detection assays in tissue and blood which can expeditiously and accurately detect colon cancer in both developed and underdeveloped regions of the world, enabling optimal use of limited resources in low- and middle-income countries.

**Supplementary Information:**

The online version contains supplementary material available at 10.1186/s13148-021-01206-2.

## Background

Colorectal cancer (CRC) is the third most commonly diagnosed malignancy and the second most common cause of cancer deaths worldwide. In 2020, 1.93 million people suffered from CRC globally, causing 935,000 deaths [1, 2]. Improvements in screening, advanced treatment strategies and changes in risk factor patterns, such as reduced smoking [3, 4], have stabilized or decreased trends in CRC mortality and incidence in western European countries and the United States [5–7]. On the other hand, CRC incidence and mortality rates have continued to rise in many low- and middle-income countries such as some Eastern European countries and diverse populations in Latin America and Asia [5–7]. Limited resources in these countries and as a result, less accessible and effective screening programs, lead to later stage at diagnosis rendering treatment more extensive and less successful [8]. The global challenge is to establish a novel, easy, quick and low-cost CRC detection method tailored especially for low resource countries facing rising trends in CRC incidence and mortality, as populations in these countries adopt a more western lifestyle [9].

The substantial risk of precancerous colorectal adenomas to progress to CRC (cumulative risk of 25.2–42.9% over 10 years) necessitates inclusion of sensitive tests to detect malignant and premalignant colorectal lesions in CRC screening programs [10, 11]. Current screening tools for CRC are mainly colonoscopy and non-invasive approaches based on detecting occult blood or cancer-specific molecular markers in stool. Colonoscopy is a highly sensitive screening test and is currently the gold standard for CRC detection. However, it is costly, invasive, involves risk of complications, and requires a skilled examiner [12]. Studies have shown that screening for CRC results in early detection enables curative treatment options, and is effective in reducing CRC death rates by 15–33% [13, 14]. Yet, global participation rates for CRC screening remain low compared to screening methods for other types of cancer, with an intercountry variability between 16 and 68.2% [15, 16]. Even in a developed country like the United States, the majority of patients presents with metastatic CRC [7, 17]. The unmet need is to develop an accurate but easy to perform, accessible, inexpensive, and minimally invasive molecular test for CRC. Such a screening test in blood or stool could help to rapidly triage patients who require follow-up to more cumbersome endoscopic approaches, and this would increase participation rates.

Few FDA-approved methylation-based liquid biopsy tests for early detection of CRC or for its use as an ancillary diagnostic have been developed, reviewed in [18]. Cologuard^®^ and Epicolon stool tests are based on a diverse panel of markers, including methylated markers, and are effective in detecting CRC [18, 19].

CpG island hypermethylation in promoter regions of tumor suppressor genes is one of the most common and earliest acquired epigenetic changes in cancer pathogenesis, including CRC [20–23]. Consequently, detection of aberrant DNA methylation in body fluids has high potential for diagnosing CRC in its early stages and monitoring disease progression and treatment response in a minimally invasive manner. DNA methylation markers for the early detection of CRC have been studied extensively; however, lack of a systematic approach in devising marker panels, limited sensitivities and/or limited specificities have rendered them inadequate for CRC screening [24, 25].

Hypermethylation of specific genetic loci can be shared among different types of cancer [26]. Previously, we identified and validated DNA methylation markers for breast cancer [27]. These highly sensitive and specific breast cancer markers, when evaluated in silico in CRC TCGA databases, also showed high potential as biomarkers for colon cancer (CC) [27]. In the current study, we carefully selected a panel of DNA methylation markers that demonstrate high levels of sensitivity and specificity for detection of colon adenocarcinoma in fresh frozen tissues by the quantitative multiplex methylation-specific PCR (QM-MSP) assay. In a pilot study, we tested these markers in cell-free plasma DNA of patients with stage IV CRC with our highly sensitive laboratory assay, cMethDNA. Our aim is for this gene marker panel to form the basis of development of a novel self-contained automated CC detection assay, similar to our prototype Breast Cancer Detection Assay (Research Use Only) [28, 29]. The Breast Cancer Detection assay prototype is run on a GeneXpert^®^ system (available throughout Africa and India), and accurately and rapidly distinguishes between cancerous and benign growths both in fine needle aspirates of the breast lesion and enlarged axillary lymph nodes [28, 29]. This novel automated CC screening technique, if applied to DNA from plasma or stool samples, has the potential to hasten cancer detection throughout the world by increasing screening participation rates due to its simplicity and through optimal risk stratification of patients who require follow-up with more invasive endoscopic techniques. It will enable optimal use of limited resources in low- and middle-income countries, which is crucial to reduce CC mortality rates globally.

## Results

### In silico study of 22 CpG loci hypermethylated in colon cancer and breast cancer

**Fig. 1:**
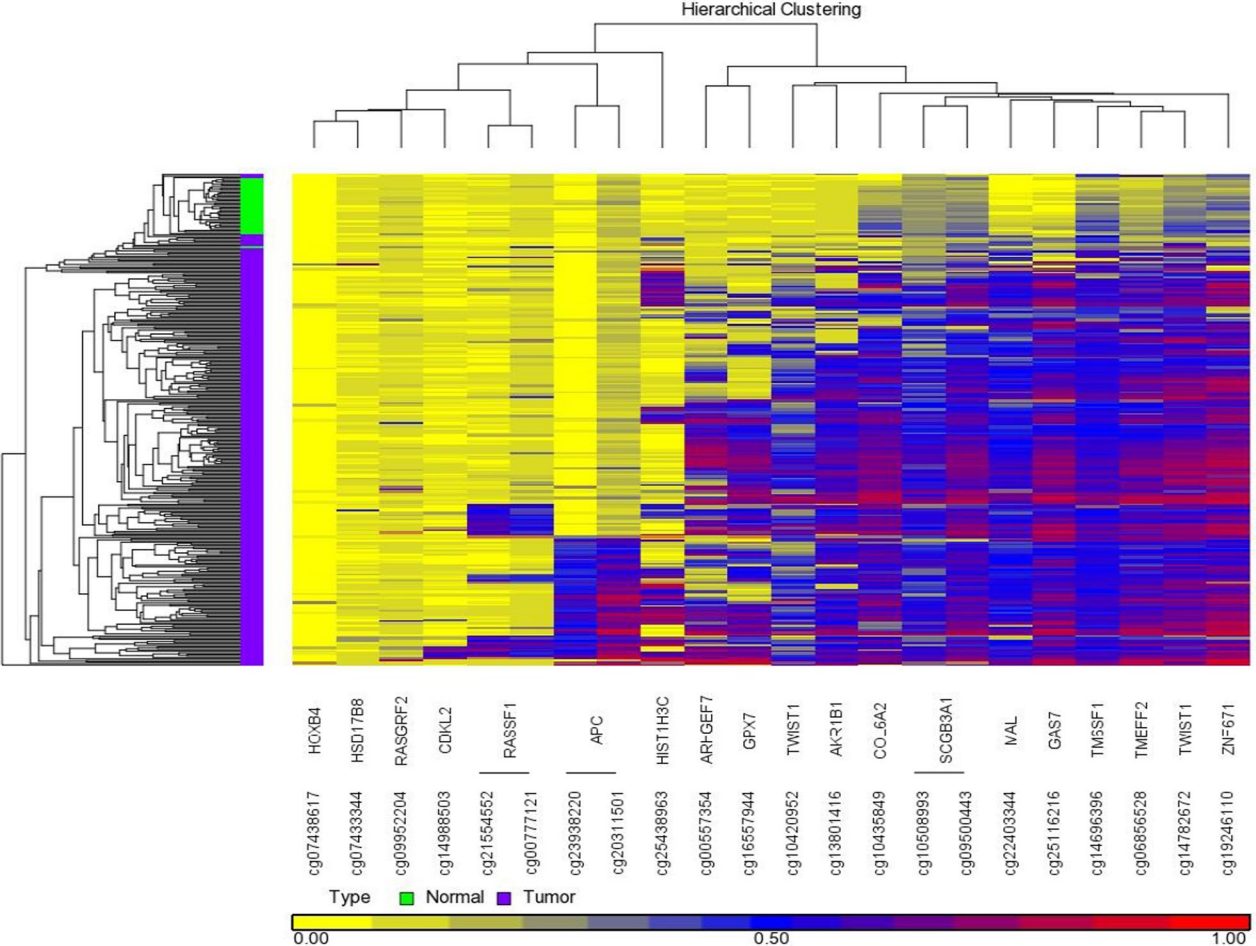
Colon adenocarcinoma (COAD)—22 CpG loci in The Cancer Genome Atlas (TCGA) Infinium HumanMethylation450 BeadChip array. In silico two-dimensional hierarchical analysis was performed to evaluate the extent of methylation among known candidate biomarkers (22 CpG probes, 18 genes, X-axis) to determine the extent of differential methylation in colon adenocarcinoma (COAD) (Y-axis, purple = carcinoma, *N* = 289; green = normal, *N* = 38). Rows and columns were clustered according to average linkage and Euclidian distance, using unadjusted β-methylation values. 13 methylated genes (the top right cluster, *APC* and *HIST1H3C*) were selected for further evaluation by QM-MSP

Based on previous studies showing high performance of methylated markers in detecting breast cancer [26], proven cancer markers were investigated for detection of CC. Using The Cancer Genome Atlas (TCGA) database, 22 CpG loci were surveyed. A cluster of 13 CpG loci (11 genes) were identified that displayed differential methylation in colon adenocarcinoma (COAD) (*N* = 289) compared to normal colon tissues (*N* = 38): *ZNF671*, *TWIST1* (two CpG loci), *TMEFF2*, *TM6SF1*, *GAS7*, *MAL*, *HIN1* (two CpG loci; *SCGB3A1*), *COL6A2*, *AKR1B1*, *GPX7*, *ARHGEF7*) (Fig. 1). *HIST1H3C* and *APC* were outside this cluster, but showed strong methylation in some CC samples and were therefore included in our panel of 13 genes for further investigation.

### Quantitation of DNA methylation in colon tissues, training set

**Fig. 2:**
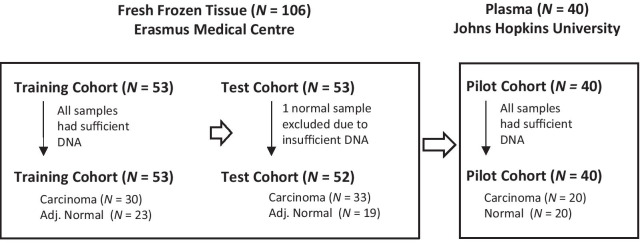
Study design. Fresh frozen samples of colon carcinoma and normal colon tissues were randomized into training and test sets matched for patient age. Thirteen markers selected in Fig. 1 were quantitatively evaluated for DNA methylation using the QM-MSP assay in samples from the training set (*N* = 53). A subset of markers was then selected based on high methylation in carcinoma and low methylation in normal tissue adjacent to tumor (Adj. Normal). A 13-gene and a 6-gene panel were evaluated in the test cohort (*N* = 52). To explore the possibility that these markers were useful in liquid biopsy, a pilot study of plasma samples from colorectal carcinoma patients (*N* = 20) and normal individuals (*N* = 20) was tested for the presence of cell-free plasma DNA using the cMethDNA assay

**Fig. 3:**
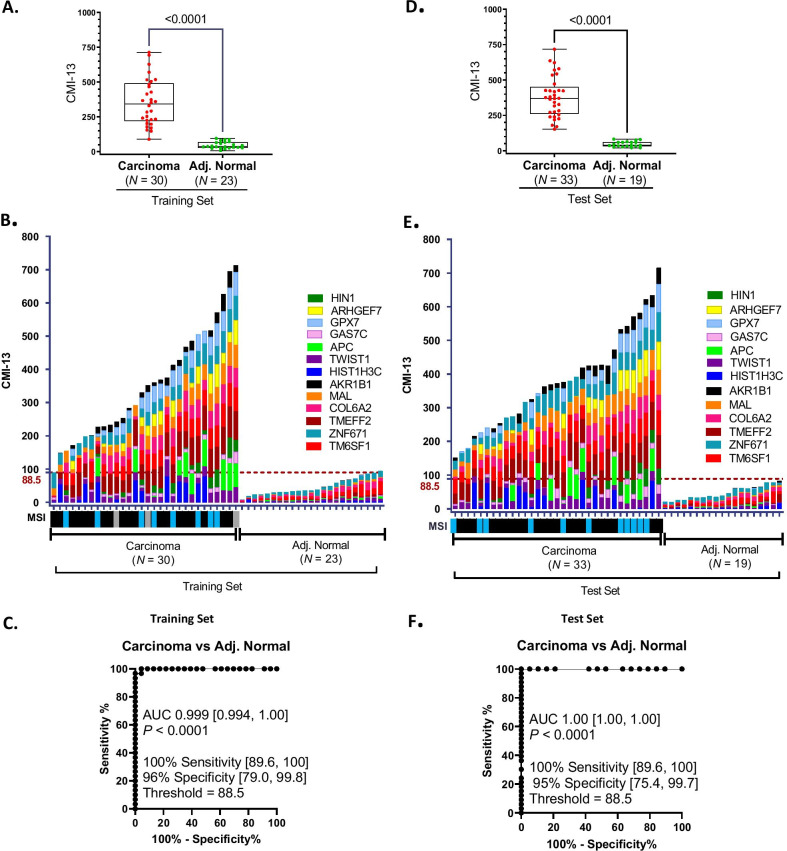
Detection of colon adenocarcinoma using cumulative methylation of 13 methylated markers. Fresh frozen colon carcinoma and adjacent normal (Adj. Normal) tissues were analyzed for cumulative methylation using QM-MSP. A panel of 13 genes, a subset of the 22 CpG loci (18 genes) indicated in Fig. 1, were evaluated. The percent methylation (%M) of each gene and the cumulative methylation index (CMI; the sum of %M for all genes in the panel) were determined for each sample. Box–whiskers plots. In training set (**A**) and test set (**D**) significantly higher methylation was observed in carcinoma compared to adjacent normal tissues (*P* < 0.0001; Mann–Whitney). Histogram plots. Data from samples in the training set (**B**) and test set (**E**) are plotted. For each sample (X-axis), the height of the histogram bar indicates the level of cumulative methylation (Y-axis), each colored segment represents an individual gene, and the size of the segment is proportional to the %M of that gene. Microsatellite instability (MSI) status for each carcinoma is indicated by the bar map below the X- axis: blue (microsatellite unstable), black (microsatellite stable), and gray (unknown). Receiver operating characteristic (ROC) curves. The ROC curve from the training set **(C)**, carcinoma versus normal control, identified a laboratory threshold maximized for sensitivity for detecting carcinoma at a specificity > 90%. This threshold (CMI = 88.5) was locked and used to determine the assay sensitivity and specificity for the test set (**F**)

The study design and the number of samples analyzed in each of training and test sets of tissues, and the pilot conducted in plasma, is presented in Fig. 2. The demographic information and clinical characteristics are presented in Table 1. Individual gene methylation of the 13 candidate genes identified by in silico analysis was quantified in the training set (Table 1, Fig. 2) of fresh frozen colon tissue samples (carcinoma, *N* = 30, adjacent normal, *N* = 23) using QM-MSP [30]. Cumulative methylation index (CMI) of the 13-gene panel in the training set is shown in Fig. 3. The CMI of the panel was significantly higher in carcinomas than in normal samples (Fig. 3A; *P* < 0.0001 by Mann–Whitney). In the histogram in Fig. 3B, the height of the histogram bar indicates the level of cumulative methylation (CMI-13) in each sample, each colored segment represents an individual gene, and the size of the segment is proportional to the percent methylation (%M) of that gene. Below the histogram, available data on microsatellite instability [31] on these tumors, categorized as MSI (microsatellite unstable) or MSS (microsatellite-stable), are presented as a bar map. No pattern of correlation between MSI, MSS, and extent of methylation was observed in the histogram. In the training set, receiver operating characteristic (ROC) analysis established the laboratory methylation threshold (CMI = 88.5, dotted line) that best distinguished CC from normal, maximizing sensitivity while retaining a minimum of 90% specificity. For detection of CC versus adjacent normal tissue in the training set, the sensitivity was 100% [95% CI 89.6, 100] and the specificity was 96% [95% CI 79.0, 99.8] with an area under the curve (AUC) of 0.999 [95% CI 0.994, 1.00; *P* < 0.0001] (Fig. 3C).

**Table 1:** Clinical characteristics of cohorts used for training, testing and pilot validation of the methylation biomarkers

Tissue	Training set	Test set	Plasma	Pilot set
Colon ca. (N)	30	33	Colorectal ca. (N)	20
Age at surgery (years)			Age (years)	
Mean	69	69	Mean	56
Median	68	69	Median	58
Range	55–86	56–85	Range	40–68
Sex (N)			Sex (N)	
Male	11	19	Male	11
Female	19	14	Female	9
Tumor cellularity (N)				
0–40%	2	1		
≥ 40–70%	17	16		
≥ 70%	11	16		
Stage			Stage	
I	7	11	I	0
II	11	15	II	0
III	12	7	III	0
IV	0	0	IV	20
Location			Location of Primary	
Colon ascendens	14	18	Colon ascendens	9
Colon transversum	1	2	Colon transversum	0
Colon descendens	3	3	Colon descendens	2
Sigmoid	11	10	Sigmoid	7
Rectum	0	0	Rectum	2
Not available	1	0	Not available	0
% Treated (N)				
Neoadjuvant	0 (0)	0 (0)		
Adjuvant	38.7 (12)	20.6 (7)		
Normal, adjacent (N)	23	20	Normal (N)	20
Age at surgery (years)			Age (years)	
Mean	66	65	Mean	70
Median	65	65	Median	70
Range	55–86	56–85	Range	26–97
Sex (N)			Sex (N)	
Male	11	10	Male	13
Female	12	10	Female	7

The performance of each of the 13 candidate markers in the training set was also analyzed individually as shown in Table 2 and as box–whiskers plots and Mann–Whitney analyses (Additional file 1: Fig. S1), and evaluated for ROC, Positive Predictive Value (PPV), Negative Predictive Value (NPV) and Accuracy (Additional file 2: Table S1). With the exception of *HIST1H3C* and *APC,* each individual gene was significantly more methylated in CC compared to adjacent normal tissues and also showed high level of performance by ROC AUC, ranging from 0.772 [ARHGEF7, 95% CI 0.646, 0. 899] to 1.000 [MAL, 95% CI 1.000, 1.000)]. Detection sensitivity ranged from 53.3 [ARHGEF7, 95% CI 36.1, 69.8] to 100% [MAL, 95% CI 88.6, 100.0], and specificity was 100% for 12 of 13 markers at the specified thresholds. Assuming a population prevalence approximated at 1% for colon carcinoma, PPV and NPV were 100% for the majority (8 of 13) of individual markers (Additional file 2: Table S1).

**Table 2:** Differential methylation in individual biomarkers

Tissue	*TMEFF2*		*GPX7*		*MAL*		*ARHGEF7*		*TWIST1*	
	Ca	No	Ca	No	Ca	No	Ca	No	Ca	No
Number of samples	30	23	30	23	30	23	30	23	30	23
%M per sample										
Minimum	0	0	0	0	9	0	0	0	6	1
25th percentile	40	0	1	0	21	1	0	0	14	3
Median	59	1	33	0	33	2	8	0	18	4
75th percentile	75	3	53	0	49	3	33	0	28	6
Maximum	94	11	72	2	71	7	75	2	50	11
*P* value (Ca. vs. No.)	< 0.0001		< 0.0001		< 0.0001		< 0.0001		< 0.0001	

Tissue	*AKR1B1*		*HIN1*		*GAS7C*		*TM6SF1*		*ZNF671*	
	Ca	No	Ca	No	Ca	No	Ca	No	Ca	No
Number of samples	30	23	30	23	30	23	30	23	30	23
%M per sample										
Minimum	0	0	0	0	0	0	0	1	0	2
25th percentile	1	0	0	0	6	0	35	5	25	7
Median	16	0	5	0	9	1	47	10	39	13
75th percentile	21	0	18	0	12	2	58	15	49	16
Maximum	52	1	61	0	36	4	70	40	72	20
*P* value (Ca. vs. No.)	< 0.0001		< 0.0001		< 0.0001		< 0.0001		< 0.0001	

Tissue	*COL6A2*		*APC*		*HIST1H3C*	
	Ca	No	Ca	No	Ca	No
Number of samples	30	23	30	23	30	23
%M						
Minimum	0	1	0	0	0	0
25th percentile	12	4	0	1	0	0
Median	34	8	0	2	1	1
75th percentile	52	10	58	2	55	3
Maximum	66	23	92	7	78	8
*P* value (Ca. vs. No.)	< 0.0001		0.2662		0.3959	

Ca, Carcinoma; No, adjacent normal tissue; *P* value for Mann–Whitney test

Methylation levels of 13 individual markers in colon carcinoma lesions – Descriptive Statistics. Fresh frozen tissues in the Training set were assayed for DNA methylation by QM-MSP. Non-parametric analyses of carcinoma (Ca) and adjacent normal (No) tissues indicated the percentiles of methylation and extent of differential methylation (Mann-Whitney *P*-value) between tissue types. *TM6SF1*, *ZNF671*, *COL6A2* showed the highest background in adjacent normal colon tissues as observed at the 75th percentile. The other genes were ranked by percent methylation (%M) at the 75th percentile among carcinoma samples: *TMEFF2* > *GPX7* > *MAL* > *ARHGEF7* > *TWIST1* > *AKR1B1* > *HIN1* > *GAS7*. Additional file 1: Fig. S1 and Additional file 2: Table S1 show the box whiskers plots and performance analyses for these data

### Testing the 13-gene panel of methylated markers

The 13-gene panel was then tested in an independent test set of fresh frozen tissues (carcinoma, *N* = 33, adjacent normal, *N* = 19, Fig. 2) using QM-MSP. Similar to the training set samples (Fig. 3A), the CMI of the panel was significantly higher in carcinomas than in normal samples (Fig. 3D; *P* < 0.0001 by Mann–Whitney) and visually represented as a histogram (Fig. 3E). Below the histogram, available data on microsatellite instability [31] on these tumors, categorized as MSI (microsatellite-unstable) or MSS (microsatellite-stable), is presented as a bar map. Similar to the training set, no pattern of correlation between MSI, MSS, and extent of cumulative methylation for 13 genes was observed in the tumor panel. As shown in Fig. 3F, using the laboratory threshold for methylation established in the training set (CMI-13 = 88.5) for the detection of CC versus adjacent normal tissue, the assay achieved a sensitivity of 100% [95% CI 89.6, 100] and a specificity of 95% [95% CI 75.4, 99.7] with an AUC of 1.00 [95% CI 1.00, 1.00; *P* < 0.0001].

We also investigated a correlation between MSI and methylation in the combined training and test set of colon carcinoma tissue samples (*N* = 60). No correlation was observed between MSI, MSS and extent of cumulative methylation for the 13-gene panel (*P* = 0.237, Mann–Whitney). However, analyzed individually, among the 13 markers %M was significantly higher in MSI (*N* = 20) compared to MSS (*N* = 40) tumors for *ARHGEF7* (*P* < 0.0001), *HIN1* (*P* < 0.0001), *GPX7* (*P* = 0.0003), and *COL6A2* (*P* = 0.0010) (Additional file 1: Fig. S6).

### Selection of a minimal 6- gene panel for detection of CC

**Fig. 4:**
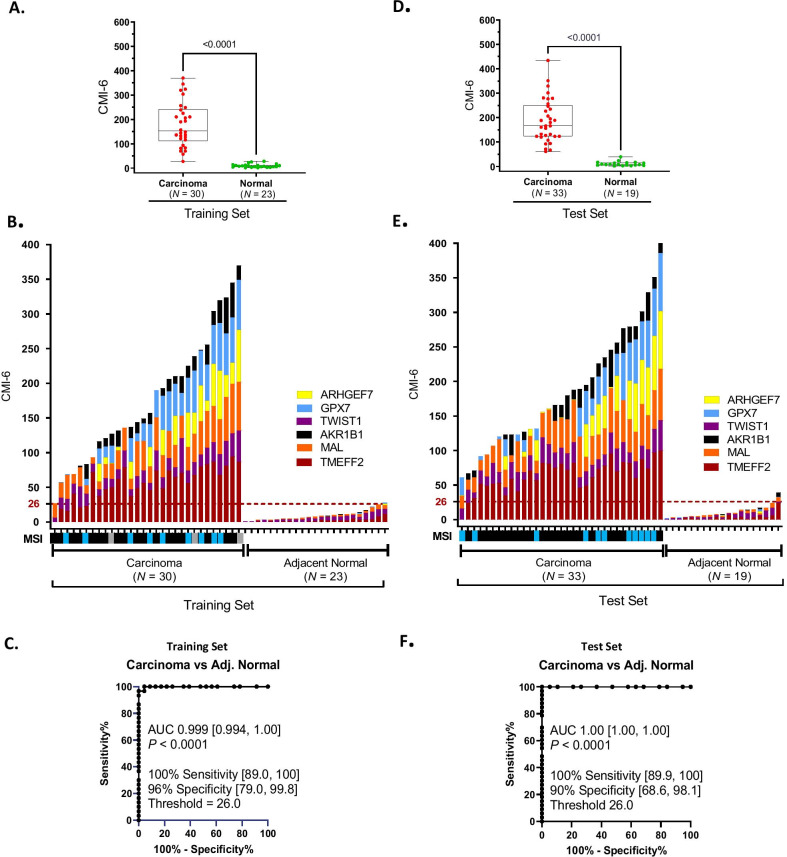
Detection of colon adenocarcinoma with a 6-gene marker panel. To identify a minimal marker panel, cumulative DNA methylation data using QM-MSP from fresh frozen tissues of colon adenocarcinoma and adjacent normal tissues were re-analyzed. A panel of 6 genes, a subset of the 13-gene panel shown in Fig. 3, was selected using criteria described in Table 2 legend. The percent methylation (%M) of each gene, and the cumulative methylation index (CMI; the sum of %M for all genes in the panel) were determined for each sample. Box–whiskers plot: In training (**A**) and test samples (**D**) significantly higher methylation was observed in samples of carcinoma compared to adjacent normal tissues (*P* < 0.0001; Mann–Whitney). Histogram plot: Data from samples in the training set (**B**) and Test set (**E**) are plotted**.** For each sample (X-axis), the height of the histogram bar indicates the level of cumulative methylation (Y-axis), each colored segment represents an individual gene, and the size of the segment is proportional to the %M of that gene. Microsatellite instability (MSI) status for each carcinoma is indicated by the bar map below the X-axis: blue (microsatellite unstable), black (microsatellite stable), and gray (unknown). Receiver operating characteristic (ROC): The ROC curve from the training set (**C**), carcinoma versus normal control, identified a laboratory threshold maximized for sensitivity for detecting carcinoma at a specificity > 90%. This threshold (CMI = 26.0) was locked and used to determine the assay sensitivity and specificity for the test set (**F**)

To reduce the 13-gene panel to a minimal size and still maintain high sensitivity and specificity, we used a two-step analytical approach involving statistical ranking and Mann–Whitney analyses, as described in Methods. First, *HIST1H3C* and *APC* were discarded based on their inability to significantly differentiate between CC versus adjacent normal colon tissues in the training set (Table 2, Additional file 1: Fig. S1 and Additional file 2: Table S1). *TM6SF1*, *ZNF671*, and *COL6A2* were then eliminated based on high background methylation (≥ 10% M per gene per sample, Additional file 1: Fig. S1) in normal colon tissues as observed at the 75th percentile of methylation (Table 2). This left 8 markers for consideration in the minimal marker panel for tissue. We ranked the markers in descending order of highest methylation at the 75th percentile of cumulative methylation among CC samples and found the methylation order was *TMEFF2* > *GPX7* > *MAL* > *ARHGEF7* > *TWIST1* > *AKR1B1* > *HIN1* > *GAS7* (Table 2). The top six markers were selected for a 6-gene marker panel. The 6-gene panel was first evaluated in the training set. The CMI of the panel was significantly higher in CC compared to normal samples as shown by Mann–Whitney analysis (*P* < 0.0001), and in the histogram (Fig. 4A, B). ROC analysis determined the laboratory methylation threshold (CMI = 26.0) that best distinguished CC versus normal tissue, maximized for sensitivity while retaining at least 90% specificity. For detection of CC (Fig. 4C), the sensitivity was 100% [95% CI 89.0, 100] and specificity was 96% [95% CI 79.0, 99.8] at a threshold of 26.0 CMI units, and the test achieved an AUC of 0.999 [95% CI 0.994, 1.00; *P* < 0.0001].

In the test set, for the 6-gene panel, CC tissues were significantly more methylated than normal tissues as shown by Mann–Whitney analysis (*P* < 0.0001) and histogram (Fig. 4D, E, respectively). Based on the laboratory methylation threshold set in the training set (CMI = 26.0), in the test set the detection sensitivity of the 6-gene panel for CC was 100% [95% CI 89.9, 100] and specificity was 90% [95% CI 68.6, 98.1] with an AUC of 1.00 [95% CI 1.00, 1.00; *P* < 0.0001] (Fig. 4F).

### Investigating the methylated gene panel in plasma from individuals with advanced colorectal carcinoma (CRC)

**Fig. 5:**
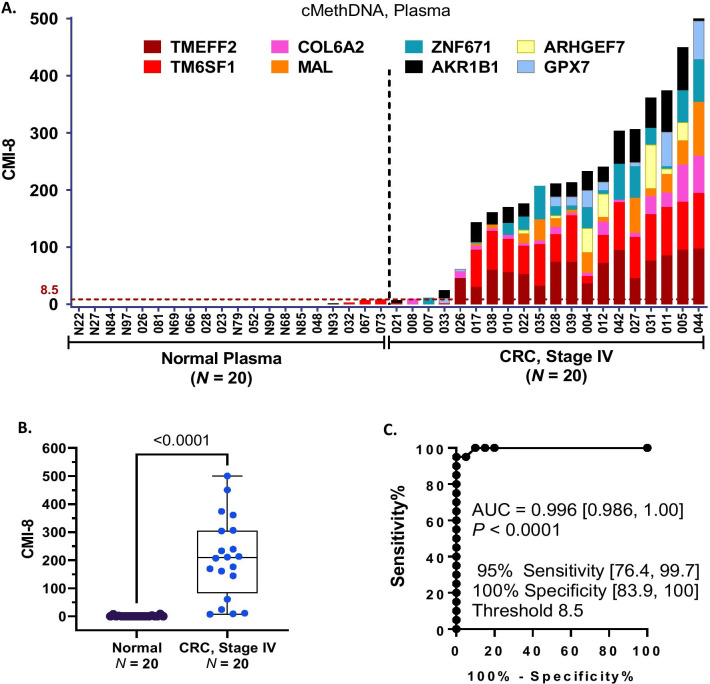
Detection of stage IV CRC in liquid biopsy. A pilot study was performed on plasma (300 μl) from 20 colorectal carcinomas (CRC stage IV) and 20 normal individuals. Using cMethDNA, a subset of nine markers found hypermethylated in colon carcinoma tissue (Fig. 1, Table 2) was assayed for circulating cell-free DNA methylation. The *TWIST1* marker failed due to technical reasons so it was excluded; the results from the 8-gene panel are shown. **A** Histogram plot. For each sample (X-axis), the height of the histogram bar indicates the level of cumulative methylation (Y-axis), each colored segment represents an individual gene, and the size of the segment is proportional to the %M of that gene. **B** Box–whiskers plot. Comparing CRC versus normal control plasma, significantly higher cumulative methylation of the 8-gene panel was observed in stage IV CRC (X-axis; Mann–Whitney, *P* < 0.0001). **C** Receiver operating characteristic (ROC). The 8-gene panel detected stage IV CRC versus normal controls with a sensitivity of 95% and specificity of 100% (AUC 0.996, *P* < 0.0001) at a threshold of 8.5 CMI. Additional file 1: Figs. S2, S3 show performance of the individual 8 markers in plasma using box–whiskers plots, Mann–Whitney and ROC analyses. Additional file 1: Fig. S4 shows the performance of the same panel in tissue using QM-MSP

The ultimate goal of our studies is to develop an automated method for detecting gene methylation in both tissue and blood, similar to the assays we are developing for breast cancer [28, 29]. Therefore, as our first attempt in liquid biopsy of CRC, we tested plasma (300 μl) of stage IV CRC patients (carcinoma *N* = 20 and normal *N* = 20) using the cell-free circulating methylated DNA (cMethDNA) assay with the 8-gene subset *TMEFF2*,* COL6A2*,* ZNF671, ARHGEF7*,* TM6SF1*,* MAL*,* GPX7*, and *AKR1B1*. *TM6SF1*, *ZNF671*, and *COL6A2* were included in this liquid biopsy panel despite the high background observed in normal colon tissue (Table 2 and Additional file 1: Fig. S1). This choice was based on our recent observations in related liquid biopsy studies that *TM6SF1*, *ZNF671*, and *COL6A2* display negligible background of methylation in plasma and serum of normal individuals. Additionally, the high methylation frequency of these markers in colon cancer tissue (Table 2, Fig. 3) rendered them eligible for inclusion in a panel for further testing. Although easily measured in tissue, *TWIST1* analysis in circulating DNA presented technical problems of reproducibility; therefore, it was excluded from further analysis in plasma. With the selected subset of 8 genes, we observed that circulating DNA was significantly more methylated in CRC plasma compared to normal plasma, as shown for individual genes (Additional file 1: Figs. S2, S3) and cumulatively for the panel of all 8 genes (Fig. 5A–C). ROC analysis determined the laboratory methylation threshold that best distinguished CRC versus normal plasma, maximized for sensitivity while retaining at least 90% specificity. At a threshold of 8.5 CMI, the detection sensitivity was 95% [95% CI 76.4, 99.7] and the specificity was 100% [95% CI 83.9, 100] achieving an AUC of 0.996 [95% CI 0.986, 1.00; *P* < 0.0001]. Performance of this 8-gene liquid biopsy panel was also evaluated in tissue. In 106 fresh frozen samples (Training and test tissue sets combined), colon carcinoma tissues were significantly more methylated than normal tissues (*P* < 0.0001, Mann–Whitney; Additional file 1: Fig. S4 A, B). For carcinoma, the sensitivity was 100% [95% CI 94.3, 100] and specificity was 100% [95% CI 91.6, 100] at a threshold of 73.5 CMI, achieving an AUC of 1.0 [95% CI 1.00, 1.00; *P* < 0.0001] (Additional file 1: Fig. S4C). This tissue CMI threshold (CMI = 73.5) was higher than the plasma threshold (CMI = 8.5) for the same markers. These results indicate that the 8-gene marker set has higher background methylation in normal tissue compared to normal plasma.

**Fig. 6:**
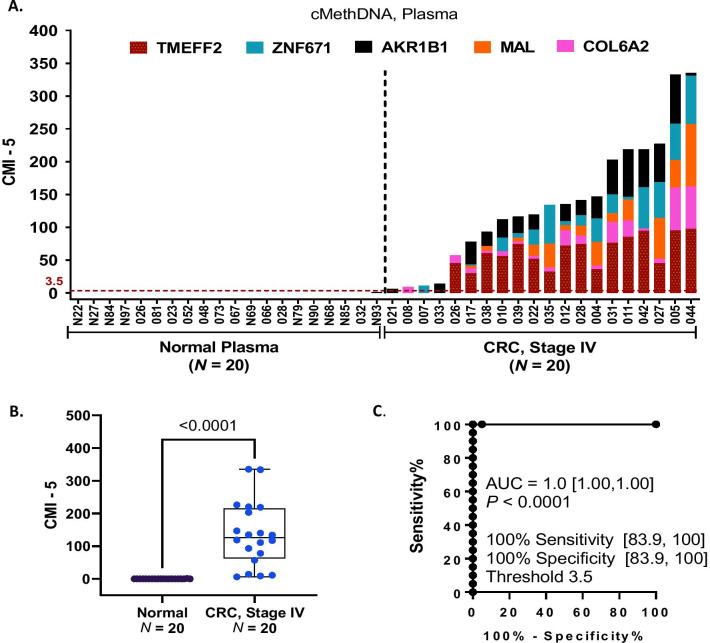
Methylated 5-gene marker panel accurately detects stage IV CRC in plasma. To identify a minimal marker subset of the 8-gene panel, cMethDNA results were re-analyzed using a subset of 5 of 8 plasma markers after selecting for individual plasma markers with the least background in normal and the highest methylation in CRC plasma (Additional file 1: Figs. S2, S3). **A** Histogram plot. For each sample (X-axis), the height of the histogram bar indicates the level of cumulative methylation (Y-axis), each colored segment represents an individual gene, and the size of the segment is proportional to the %M of that gene. **B** Box–whiskers plot. Comparing CRC versus normal control plasma, significantly higher cumulative methylation of the 5-gene panel was observed in stage IV CRC (X-axis; Mann–Whitney, *P* < 0.0001). **C** Receiver operating characteristic (ROC). The 5-gene panel, with negligible methylation in normal plasma identified samples from patients with stage IV CRC versus normal controls with a sensitivity of 100% and specificity of 100% (AUC 1.0, *P* < 0.0001) at a threshold of 3.5 CMI. Additional file 1: Fig. S5 shows the performance of the same panel in tissue using QM-MSP

Minimizing the number of markers when developing an automated liquid biopsy method, theoretically, has several advantages. Fewer markers enables the assay to be cost effective and it may lead to lower background in normal plasma resulting in greater specificity. Re-analyzing the data using a 5-gene subset of the 8 genes, *TMEFF2, ZNF671, AKR1B1, MAL,* and *COL6A2,* yielded very significant difference in methylation in plasma from patients with CRC compared to normal (Fig. 6A, B) and achieved an AUC of 1.0 [95% CI 1.00, 1.00; *P* < 0.0001]. This combination sensitively detected 100% [95% CI 83.9, 100] of the samples with a specificity of 100% [95% CI 83.9, 100] at a threshold of 3.5 CMI (Fig. 6C).

Evaluating the same 5-gene panel in tissue from the training and tests sets, we observed higher CMI in carcinoma compared to normal tissues (Additional file 1: Fig. S5A, S5B). The detection sensitivity of the 5-gene panel for carcinoma was 100% [95% CI 94.3, 100], and specificity was 100% [95% CI 91.6, 100] using a laboratory threshold of 58.5 CMI; the AUC was 1.0 [95% CI 1.00, 1.00; *P* < 0.0001] (Additional file 1: Fig. S5C). As expected, compared to normal plasma, this 5-gene panel had high background in normal tissue, mainly contributed by *ZNF671* and *COL6A2*. However, the results clearly show that these markers enhanced sensitivity without lowering specificity in liquid biopsy (Fig. 6C).

## Discussion

In this study, we adopted a systematic approach to select, train, and test methylated marker panels which performed with high sensitivity and specificity to detect colon carcinomas. The robust results of this study form a solid basis for validation of the methylated gene panels in tissue, blood, or stool. We have also presented pilot data on the successful application of these markers to liquid biopsy, where sensitivity and specificity approached 100%, with as few as 5 of 13 methylated colon carcinoma markers. We intend these studies to form the foundation for the development of a self-contained automated colon carcinoma assay. We have already demonstrated feasibility of this type of automated cancer detection approach in breast carcinoma [28, 29].

The 13-gene markers analyzed in the training set using QM-MSP allowed us to rank the performance of each gene based on their ability to distinguish between carcinoma and normal colon tissue as revealed by the Mann–Whitney tests (Table 2, Additional file 1: Fig. S1) and by performing ROC AUC, sensitivity, specificity, PPV, NPV, and other statistical analyses (Additional file 2: Table S1). The genes were tested in an independent set of fresh frozen samples first using panels of 13- and then just 6 of the 13 genes. With the minimal gene panel, using the laboratory thresholds defined in the training set, in the test set the 6-marker panel achieved an AUC of 1.00 [95% CI 1.00, 1.00] (Fig. 4). Our pilot study of blood from patients with stage IV CRC, albeit small, provided important information as to whether tissue markers would be useful in liquid biopsy. Although this small set contained plasma from 18 CC patients and only 2 rectal cancer patients, robust methylation was noted in all 20 samples. Whether the liquid biopsy markers will be equally efficient in detecting both colon and rectal adenocarcinomas remains to be determined in future studies of larger sample sets. In plasma, with an 8-gene panel the AUC was 0.996 [95% CI 0.986, 1.00] (Fig. 5). Reduced to a 5-gene panel, the AUC was 1.0 [95% CI 1.00, 1.00] with 100% sensitivity and 100% specificity at a threshold of CMI = 3.5 (Fig. 6). It is important to mention that these results were obtained by conducting the cMethDNA assay using just 300 ul of plasma. The data strongly suggest that a very small panel of carefully selected methylated markers has the ability to provide a robust assay for detection of cell-free methylated DNA of CRC origin in plasma. Of note, a high level of detection sensitivity and specificity was attained in tissue and plasma analyses comparing both cancer and normal samples, although normal tissue had higher background compared to normal plasma. On the subject of background, our findings bring home the fact that methylation markers may be highly sensitive but not specific in tissues (high background in normal tissue). Nevertheless, these may perform with a high level of both sensitivity and specificity in liquid biopsy and therefore deserve to be tested further. Tissue and plasma samples in this study were not from the same patient. Also, tissue samples were from stage I-III disease, while plasma samples were from stage IV cancer. Despite these shortcomings, the marker panels displayed a high level of accuracy (ROC AUC) in both tissue and circulating cell-free methylated DNA.

A potential relationship between microsatellite instability and hypermethylation of LINE elements, single genes or gene panels has been demonstrated [32–34]. But the data are confounded by many clinical factors such as age and treatment strategies. In the small number of MSI (N = 20) and MSS (N = 40) colon carcinomas in our study, we found statistically significant differential hypermethylation in MSI compared to MSS tumors for 4 of 13 individual markers [*HIN1*, *ARHGEF7*, *GPX7*, and *COL6A2* (*P* < 0.0001, *P* < 0.0001, *P* = 0.0003, *P* = 0.0010, respectively; Additional file 1: Fig. S6)]. Whether hypermethylation of these genes contributes to MSI directly or indirectly, or if they have prognostic importance, remains to be studied. These observations are promising and need to be substantiated in future studies on larger panels of tumors.

## Conclusions

Current low participation rates in CRC screening programs globally cause most CRC patients to present with metastasized disease. Although in its infancy, an innovative colon cancer detection method based on blood from the patient could provide a resource-responsive measure in low- and middle-income countries that are experiencing rapid increases in colon cancer incidence and mortality [5, 6]. The development of minimally invasive molecular tests for colon cancer detection has the potential to rapidly triage patients requiring follow-up with colonoscopy and rapid treatment, in the case of cancer. We propose that developing automated systems, such as the cartridge-based GeneXpert^®^ system, for detecting methylated markers in plasma will allow its widespread use in the developing world if they are accurate, easy to perform, and have a rapid turnaround time of a few hours.

In conclusion, in this study, gene marker panels achieving high levels of sensitivity and specificity for detection of colon cancer lesions have been described. The methylated colon cancer detection markers identified in this study serve as the foundation for future research incorporating these markers in an innovative automated assay detecting methylation in circulating CRC DNA in the blood.

## Methods

### Sample collections

Fresh frozen tissues were obtained from the MATCH study, a prospective multicenter cohort study from 2007 onward that includes adult patients undergoing curative surgery for stage I–III colon cancer in one of seven participating hospitals in the Rotterdam region of the Netherlands [31]. All patients provided written informed consent for the storage and use of tissue samples for research purposes and the collection of clinical data (Institutional Review Board (IRB) number MEC 2007-088). In total, 106 tissue samples were included in the current study, 63 carcinoma and 43 adjacent normal colon (Adj. N) tissue samples collected at a minimal distance of at least 1 cm from the tumor. Hematoxylin–Eosin stained sections from the frozen blocks of tumor confirmed the diagnosis of carcinoma normal colon. The tumor sections contained a minimum of 30% carcinoma cells [31]. During processing, one normal sample was excluded due to insufficient DNA, resulting in a total of 63 tumors and 42 normal colon tissues available for methylation analysis. In addition, a pilot study was conducted on a small set of samples of EDTA plasma collected at Johns Hopkins University (JHU) (*N* = 20 colorectal carcinomas; IRB00060125; *N* = 20 normal; IRB NA00033085), approved by the JHU institutional review board. Patient characteristics are provided in Table 1.

### Study design and workflow

The study design and workflow are shown in Fig. 2. Step 1 was to analyze in silico The Cancer Genome Atlas (TCGA) Illumina Infinium 450K HumanMethylation (450K HM) array database of colon adenocarcinoma (COAD) for 22 CpG loci in 18 known breast cancer detection genes [26–28]. Hierarchical cluster analysis was used to reveal a subset of candidate CpG loci differentially methylated in tumor versus normal tissue in COAD, a database consisting of 289 colon adenocarcinomas and 38 normal colon tissues. Step 2 was to confirm these in silico findings by assaying the fresh frozen colon tissue samples in a training set using an independent, quantitative methylation specific PCR platform (QM-MSP). The fresh frozen colon tissue samples were randomly assigned to either the training or the test sample set using the RANDBETWEEN Excel function, balancing the sets for patient age at time of surgery and sample size for each lesion type (carcinoma or adjacent normal). Step 3 was to select a candidate minimal marker panel (consisting of 6 markers) then to define a laboratory threshold of CM that achieved maximal sensitivity while obtaining a specificity of at least 90% detection of CC versus adjacent normal tissues. Step 4 was to assay the performance of this marker panel in the Test sample set using locked parameters defined in the training set in Step 3. Performance parameters included Mann–Whitney analyses, and receiver operating characteristic sensitivity, specificity, and AUC. Lastly, Step 5 was to evaluate the markers in circulating cell-free DNA in plasma of stage IV CC patients and healthy blood donors, using a highly sensitive quantitative multiplex methylation-specific polymerase chain reaction assay named cMethDNA [27].

### Quantitative multiplex methylation-specific polymerase chain reaction (QM-MSP)

#### Sample processing

Genomic DNA was extracted from freshly frozen tissues using two to ten 30-µm cryostat sections (5–20 mg; cellularity is described in Table 1) and processed with the NucleoSpin^®^Tissue kit (Macherey–Nagel; Bioké, Leiden, The Netherlands) according to the protocol provided by the manufacturer. The quantity and quality of the isolated DNA was established by Nanodrop and by PicoGreen. DNA fragment sizes were evaluated after agarose gel electrophoresis. Samples not showing a DNA band of at least 20 kb were excluded. Prior to PCR, sodium bisulfite-mediated DNA conversion was performed using the EZ DNA kit (ZymoResearch, Irvine, CA, USA; #D5001) according to the manufacturer’s instructions. QM-MSP was performed using the quantitative multiplex methylation-specific nested PCR method, as described in detail [30, 35]. The cMethDNA method and primer and probe sequences used in this manuscript are described in detail [27]. These QM-MSP and cMethDNA primer/probes were designed to be at or near the CpG sites encompassed by the 450K array. The QM-MSP %M for each gene was calculated using the formula:

$$\% M = \frac{\# copies\,methylated\,DNA}{{total\,\# copies\,methylated + unmethylated\,DNA}} \left( {100} \right)$$

In QM-MSP, cumulative methylation is expressed as the CMI, cumulative methylation index, the sum of %M for genes in the sample for a specific panel of markers.

#### Marker selection

QM-MSP was used in the training and test sets for marker selection and evaluation. Thirteen individual markers were assayed using DNA from fresh frozen tissues in the training cohort (of *N* = 53 samples) ;each gene in each sample was assigned a %M for the sample. Marker selection criteria required first that markers show significantly higher %M levels in CC than in normal colon tissue samples (*P* < 0.05, based on the Mann–Whitney test). Second, markers were required to have low levels of %M in normal samples, to further minimize the risk of false positives. More specifically, the 75th percentile of methylation was calculated to analyze methylation levels in normal samples. Background above ≥ 10%M was considered high for a single marker in a given sample. Last, markers were ranked in the order of methylation at the 75th percentile (Table 2) and the top six markers were picked to constitute the 6-gene marker panel. QM-MSP values for the panel were expressed as CMI. Using receiver-operating characteristic (ROC) curve analysis, the laboratory CMI threshold for the study which maximized sensitivity while retaining a minimum of 90% specificity was determined.

#### Microsatellite instability (MSI)

Existing MSI data [31] were available on the colon cancer tissue samples. For 60 samples in our study, this data (*N* = 20 MSI, *N* = 40 MSS) was used to analyze the association between MSI and DNA methylation for 13 genes using Mann–Whitney statistic.

### Statistical analysis

In silico hierarchical cluster analysis was performed using Euclidian distance measurements within Partek^®^ Genomics Suite^®^ software (Partek Inc., Chesterfield, MO). The QM-MSP methylation results were evaluated using GraphPad Prism (GraphPad Software version 9, La Jolla CA) descriptive statistics and nonparametric tests (Mann–Whitney). The methylation results were displayed as cumulative stacked histograms and box–whiskers plots. ROC analyses were used to determine the laboratory methylation threshold that best distinguished cancer from normal, optimizing for sensitivity while retaining specificity of at least 90%, reporting performance as AUC and the 95% confidence intervals in brackets. We emphasized both high sensitivity and high specificity for each of the markers, due to our future goals of automation of a colon cancer detection assay for potential clinical applications of cancer detection, and monitoring treatment response and disease recurrence in blood. Analyses were two-tailed and considered statistically significant at *P* < 0.05. Negative predictive value (NPV), positive predictive value (PPV), and accuracy were calculated according to the formulas below. Prevalence was approximated at 1% of the population.

$$\begin{aligned} \% NPV & = \frac{{specificity X \left( {1 - prevalence} \right)}}{{\left( {1 - sensitivity} \right) X prevalence + specificity X \left( {1 - prevalence} \right)}} \;\left( {100} \right) \\ \% PPV & = \frac{sensitivity X prevalence}{{sensitivity X prevalence + \left( {1 - specificity} \right) X \left( {1 - prevalence} \right)}}\; \left( {100} \right) \\ \% Accuracy & = sensitivity X prevalence + specificity X \left( {1 - prevalence} \right) \;\left( {100} \right) \\ \end{aligned}$$

## Supplementary Information

**Additional file 1:** Figures S1–S6.

**Additional file 2:** Table S1.

## Data Availability

All datasets described in the manuscript are available to be downloaded from TCGA.

## Abbreviations

CRC: Colorectal cancer
CC: Colon adenocarcinoma
TCGA: The cancer genome atlas
QM-MSP: Quantitative multiplex methylation-specific PCR
ROC: Receiver operating characteristic
AUC: ROC area under the curve
CMI: Cumulative methylation index
NPV: Negative predictive value
PPV: Positive predictive value
