# Supplementary material for: High performance methylated DNA markers for detection of colon adenocarcinoma
Source: Clin Epigenetics. 2021 Dec 13;13:218. doi: 10.1186/s13148-021-01206-2 (PMC8670296; doi:10.1186/s13148-021-01206-2)
Supplement: Supplementary file 1 — Additional file 1: Figures S1–S6. [file 13148_2021_1206_MOESM1_ESM.pdf]

High Performance Methylated DNA Markers for Detection of Colon Adenocarcinoma

Romy A. M. Klein Kranenbarg, Abdul Hussain Vali, Jan N. M. IJzermans, Thomas R. Pisanic II, Tza-Huei Wang, Nilofer Azad, Saraswati Sukumar, Mary Jo Fackler

Additional File 1
Figs. S1-S6

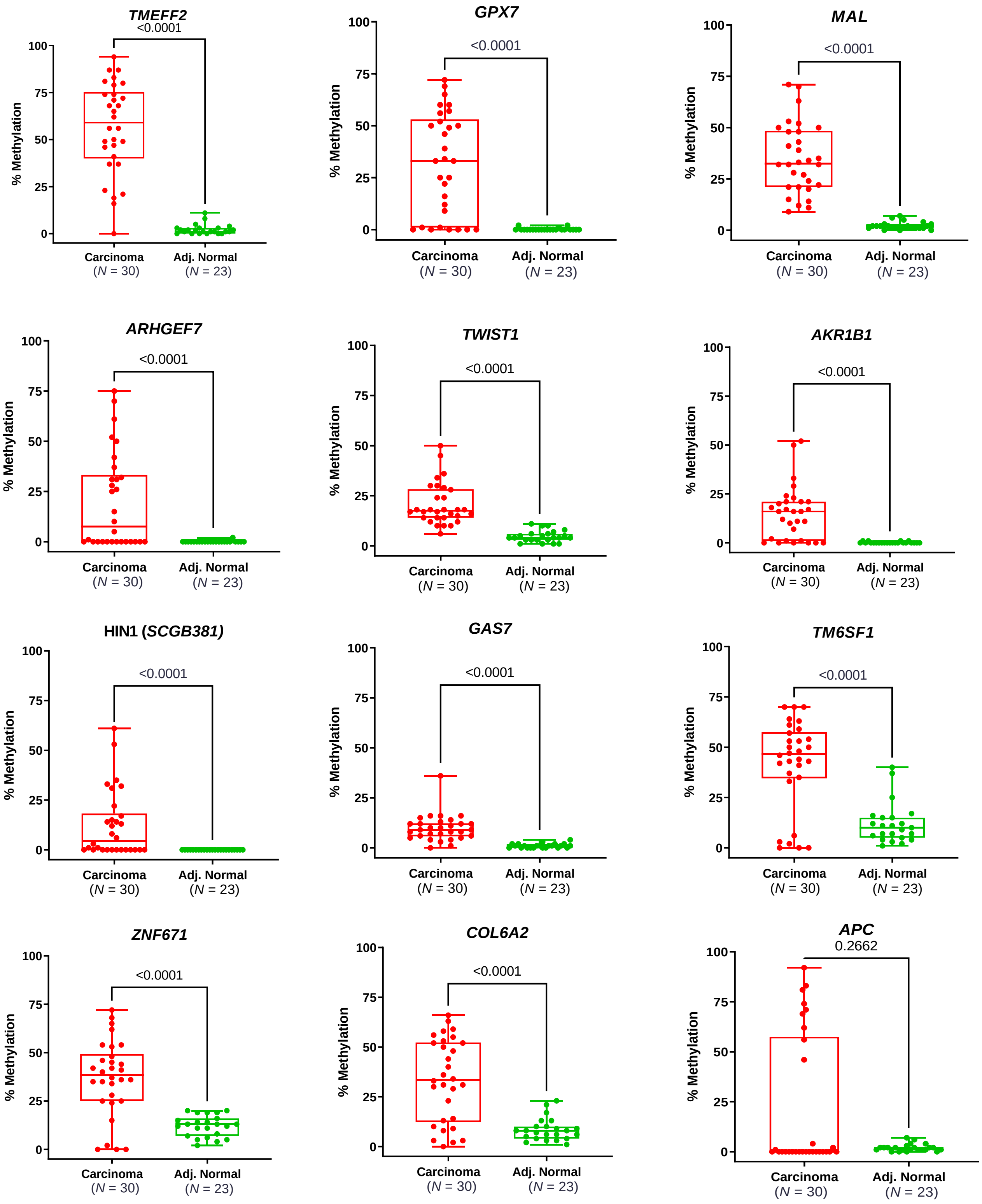

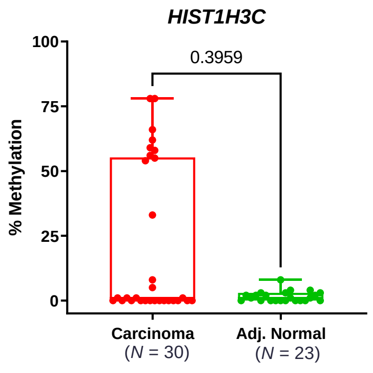

Fig. S1. Methylation of 13 individual genes in colon carcinoma and adjacent normal tissue. Fresh frozen tissues of the training set were analyzed by QM-MSP for 13 methylated DNA markers. Box Whiskers plots and Mann-Whitney analyses indicate significant ($P < 0.05$) differential methylation between adjacent (Adj.) normal colon versus colon carcinoma for all markers excepting *APC* and *HIST1H3C*. Table 2, Additional file 2: Table S1, Additional file 1: Fig. S2, respectively, show additional supporting data.

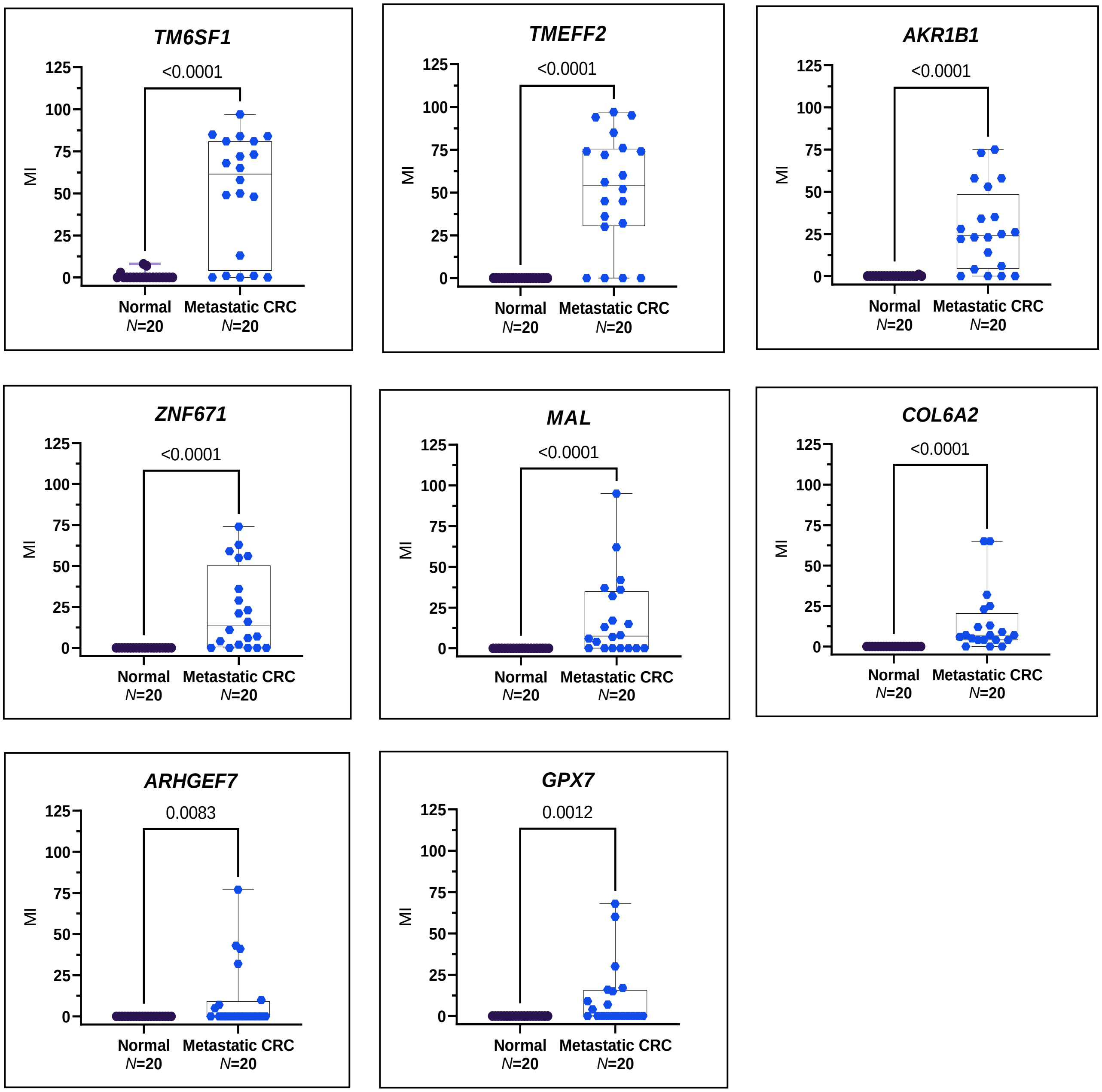

Fig. S2. Performance of 8 individual markers in detecting cell-free DNA in stage IV CRC plasma using cMethDNA. Box-whiskers plots and Mann-Whitney analyses indicate differential methylation in plasma between normal individuals versus those with stage IV CRC. All markers showed $P < 0.0001$, except *ARHGEF7* ($P = 0.0083$) and *GPX7* ($P = 0.0012$). Fig. 5, Fig. 6, and Additional file 1, Fig. S3 show supporting data.

Additional file 4

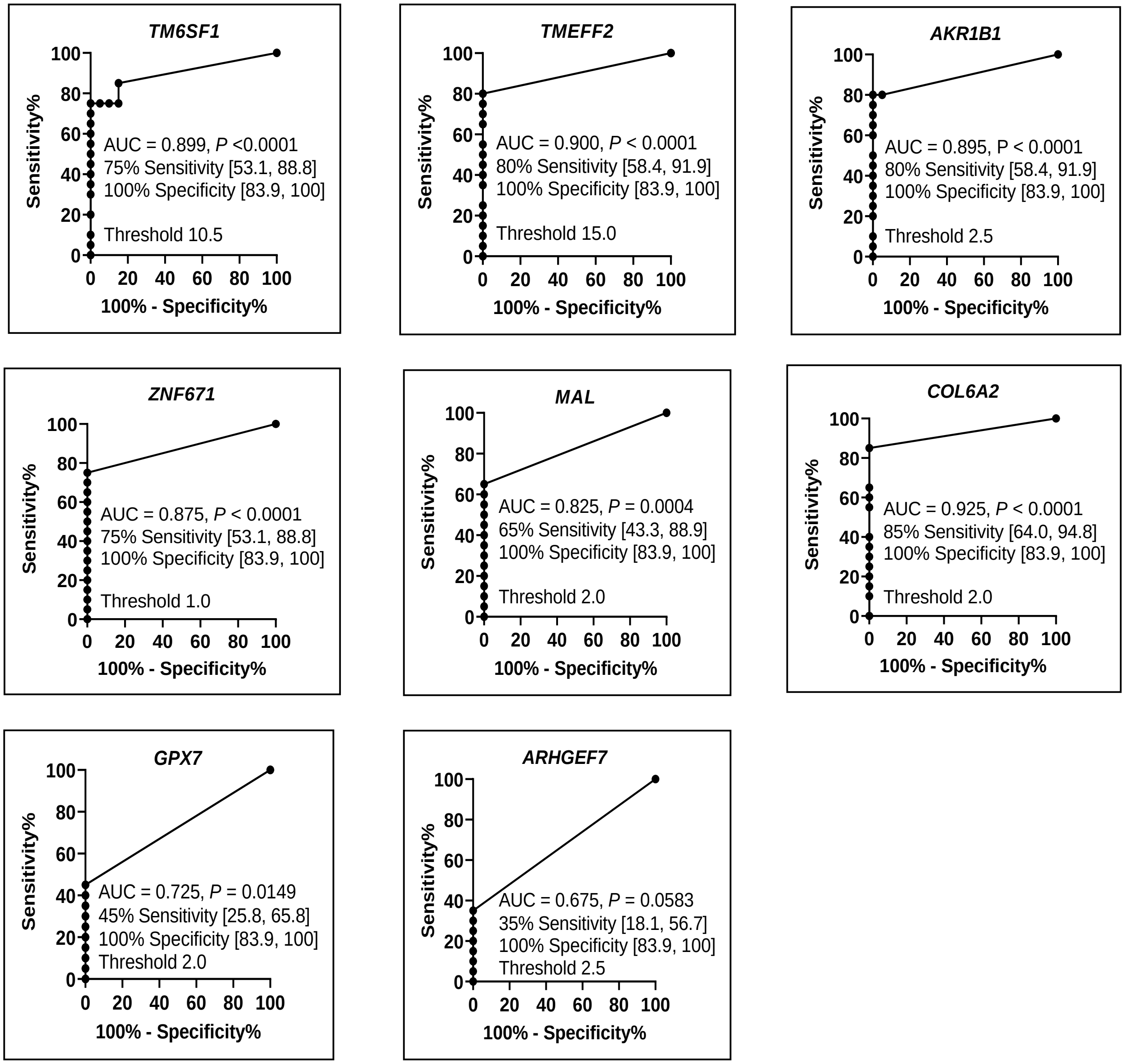

Fig. S3. ROC analyses for 8 individual methylated genes in plasma. Receiver operating characteristics (ROC) analyses was performed on cMethDNA data in plasma from normal individuals and stage IV CRC patients. Additional supporting data are shown in Fig. 5, Fig. 6 and Additional file 1, Fig. S2. Threshold MI (methylation index) was selected based on maximizing sensitivity while maintaining specificity ≥90%.

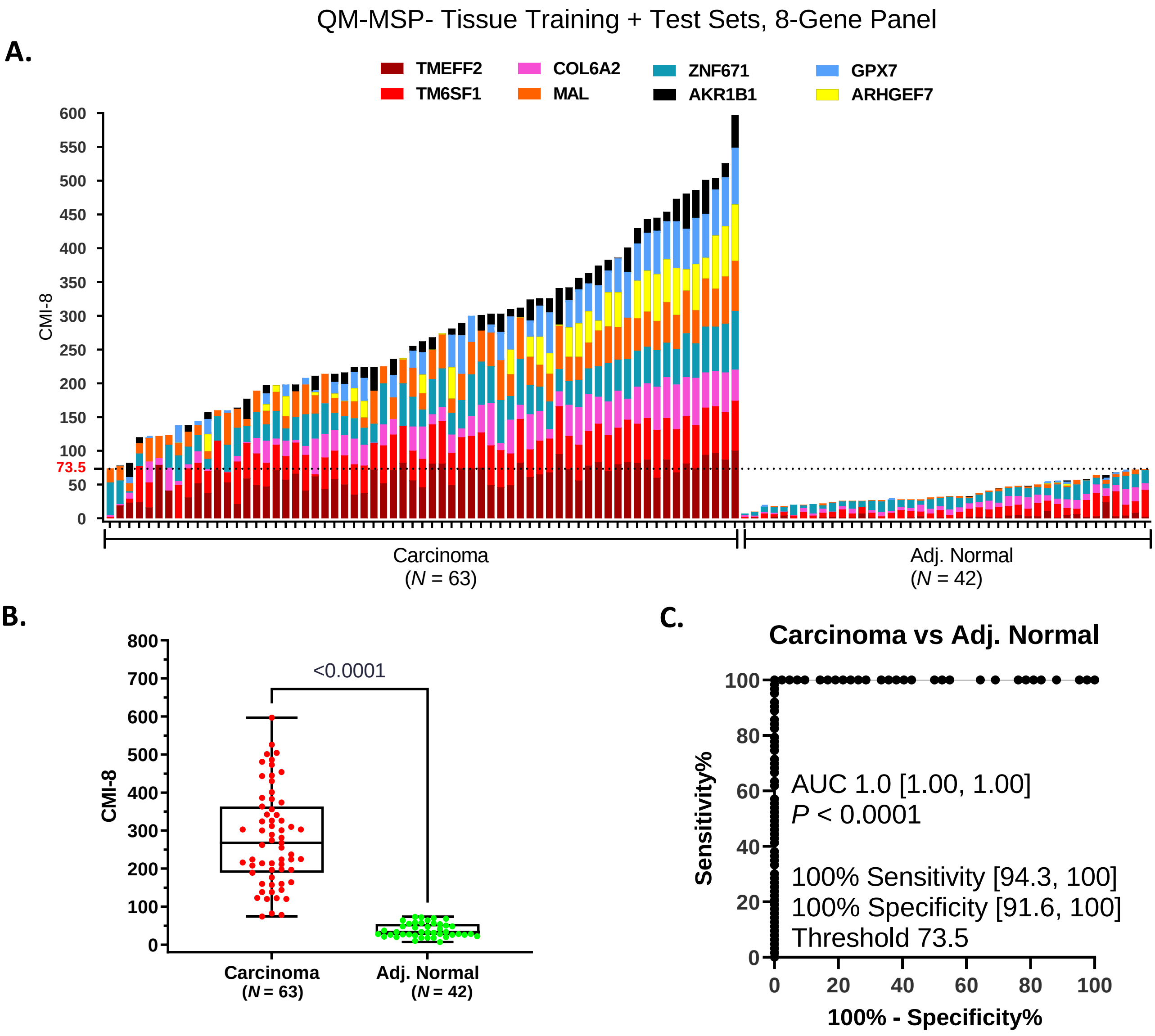

Fig. S4. Performance of the 8-marker panel, used for liquid biopsy, in colon tissue. The 8-marker panel designed for plasma (Fig. 5) was also evaluated using the QM-MSP data of combined training and test sets of tissues. **A. Histogram plot.** For each sample (X-axis), the height of the histogram bar indicates the level of cumulative methylation (Y- axis), each colored segment represents an individual gene, and the size of the segment is proportional to the percent methylation of that gene. **B. Box-whiskers plot**. In training and test samples, significantly higher methylation was observed in samples of carcinoma compared to adjacent normal tissue (P < 0.0001; Mann-Whitney). **C. Receiver operating characteristic (ROC) curves**. Using carcinoma versus adjacent normal tissue (Adj. Normal) tissue, the laboratory threshold (cumulative methylation index, CMI = 73.5) was identified, after maximizing for sensitivity; 100% sensitivity and 100% specificity with an AUC of 1.0 (P < 0.0001) was observed. This tissue CMI threshold was higher compared to the plasma threshold reported in Fig. 5, CMI = 8.5, for the same markers.

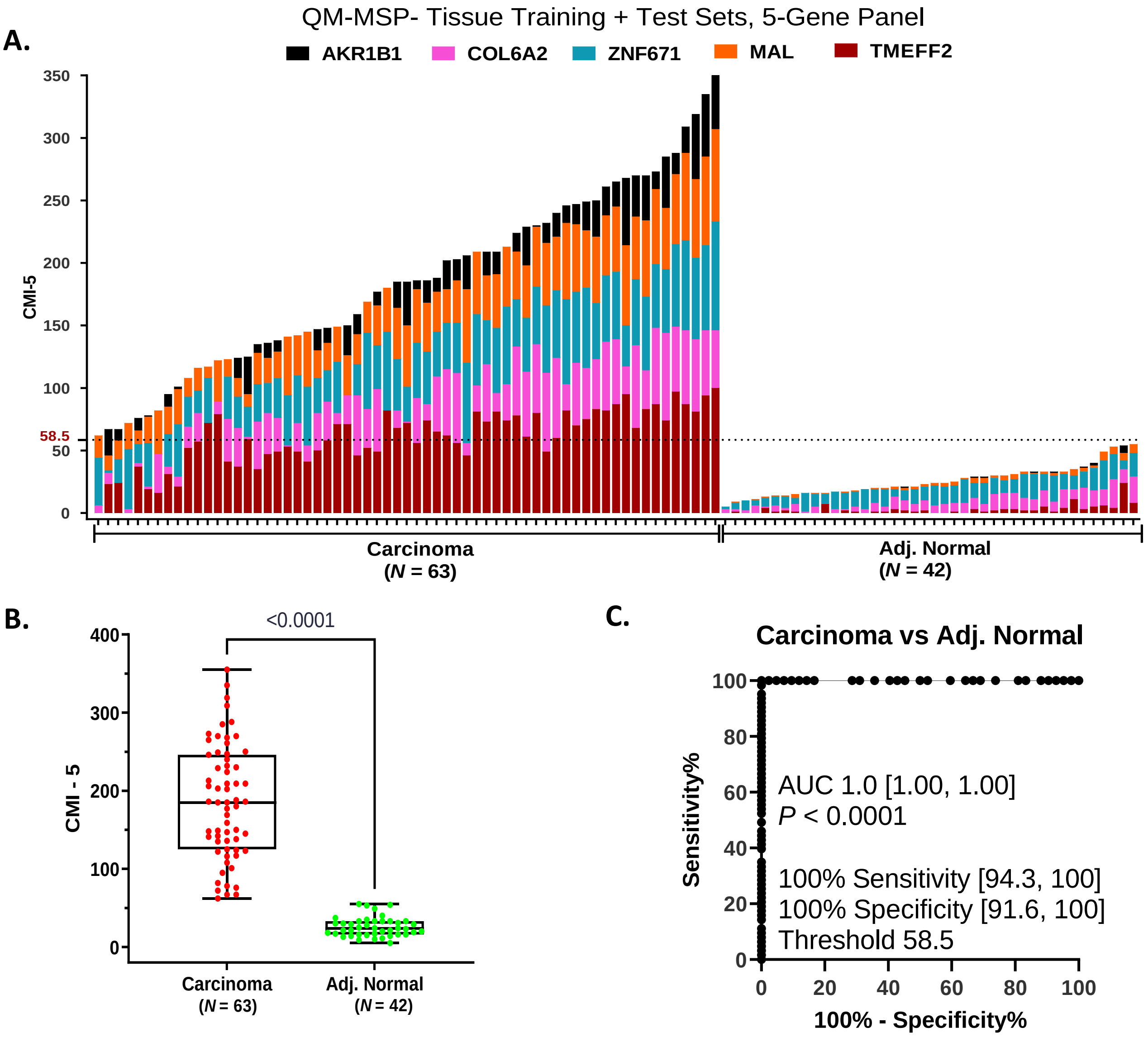

Figure S5. Performance of the 5-gene panel, used for liquid biopsy, in colon tissue. The 5-gene minimal marker panel designed for plasma (Fig. 6) was also examined using the QM-MSP data of combined training and test sets of tissues. **A. Histogram plot.** For each sample (X-axis), the height of the histogram bar indicates the level of cumulative methylation (Y-axis), each colored segment represents an individual gene, and the size of the segment is proportional to the percent methylation of that gene. **B. Box-whiskers plot**. In training and test samples, significantly higher methylation was observed in samples of colon carcinoma compared to adjacent normal (Adj. Normal) tissue (P < 0.0001; Mann-Whitney). **C. Receiver operating characteristic (ROC) curves**. Comparing colon carcinoma versus adjacent normal tissue, the laboratory threshold (CMI= 58.5) was identified, maximizing for sensitivity; 100% sensitivity and 100% specificity; an AUC of 1.0 (P < 0.0001) was observed. High background methylation in normal colon tissue, attributable primarily to *ZNF671* and *COL6A2*, was not detected in our assay of normal plasma (Fig. 6).

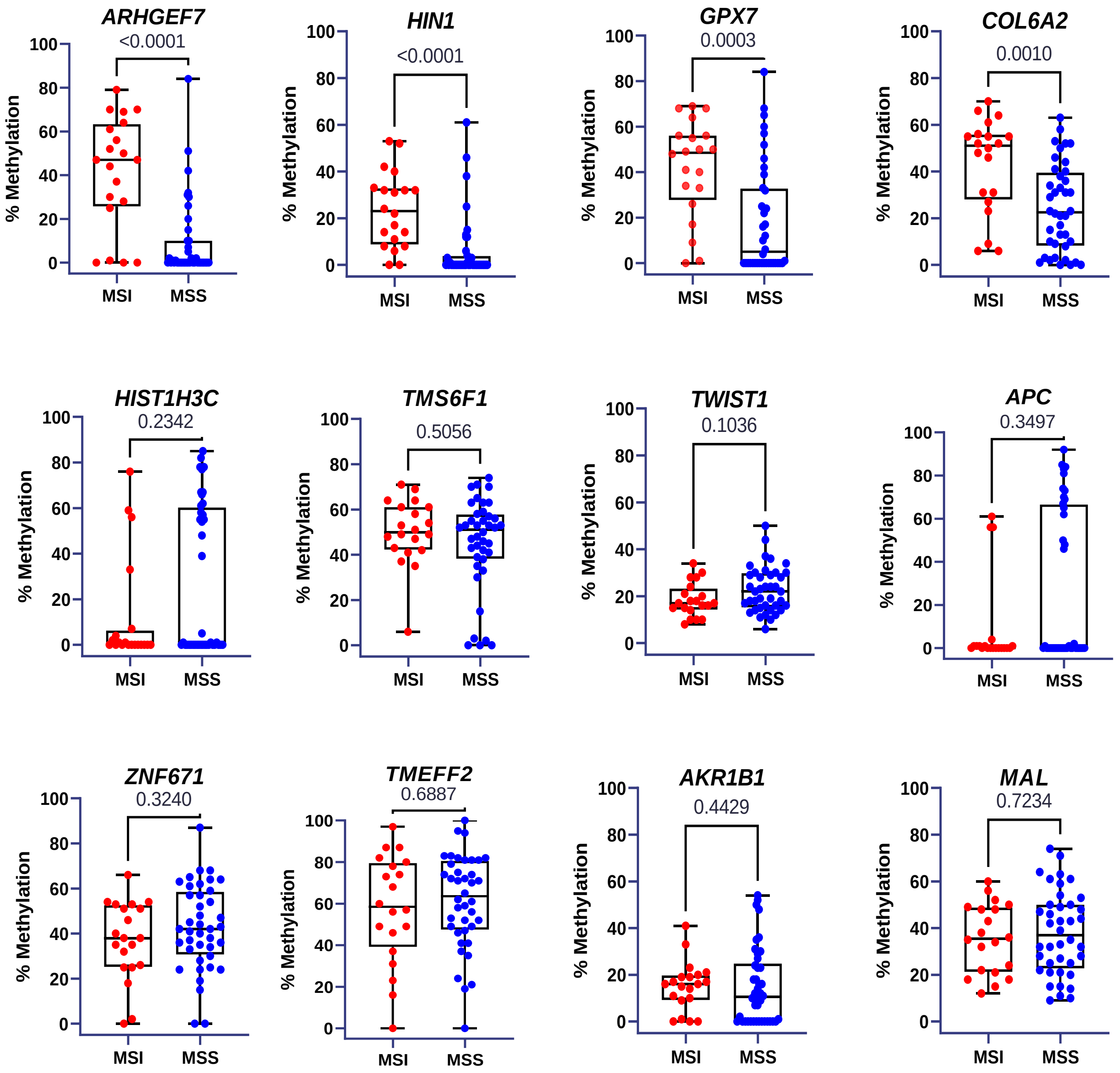

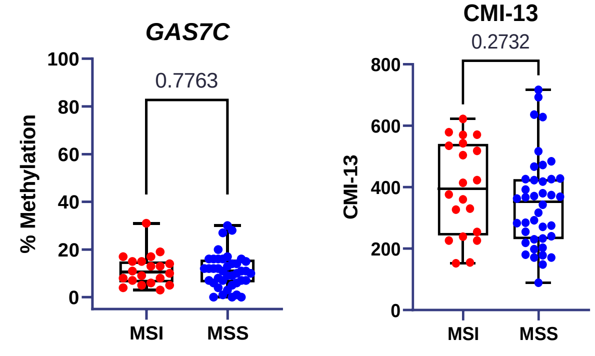

Fig. S6. Correlation between microsatellite instability and hypermethylation of individual genes in the 13-gene panel. Available MSI data [31] on the combined training and test set of tumor tissues (N = 20 MSI, N = 40 MSS) in this study were analyzed for %M versus MSI subgroup. Results are presented as box-whisker plots, and Mann-Whitney P-value. MSI: microsatellite unstable, MSS: microsatellite stable.
