# Supplementary material for: High performance methylated DNA markers for detection of colon adenocarcinoma
Source: Clin Epigenetics. 2021 Dec 13;13:218. doi: 10.1186/s13148-021-01206-2 (PMC8670296; doi:10.1186/s13148-021-01206-2)
Supplement: Supplementary file 2 — Additional file 2: Table S1. [file 13148_2021_1206_MOESM2_ESM.pdf]

Additional file 2:

TRAINING SET - Individual Marker Performance											
Marker	ROC AUC	AUC 95% CI	AUC P-value	Sensitivity %	Sens. 95% CI	Specificity %	Spec. 95% CI	ROC Threshold	PPV %	NPV %	Accuracy %
TMEFF2	0.972	0.917 - 1.000	< 0.0001	97	83.3 - 99.8	100	85.7 - 100	13.5	100	100	100.0
GPX7	0.880	0.784 - 9.760	< 0.0001	73	55.6 - 85.8	100	85.7 - 100	5.5	8	100	91.1
MAL	1.000	1.000 - 1.000	< 0.0001	100	88.6 - 100	100	85.7 - 100	8.0	100	100	100.0
ARHGEF7	0.772	0.646 - 0.899	0.0007	53	36.1 - 69.8	100	85.7 - 100	3.5	12	100	95.3
TWIST1	0.983	0.957 - 1.000	< 0.0001	87	70.3 - 94.7	100	85.7 -100	11.5	17	100	95.3
AKR1B1	0.877	0.779 - 0.974	< 0.0001	73	55.6 - 85.8	100	85.7 - 100	1.5	100	100	99.7
HIN1	0.800	0.680 - 0.920	0.0002	60	42.30- 75.4	100	85.7 - 100	0.5	100	100	99.6
GAS7C	0.955	0.895 - 1.000	< 0.0001	83	66.4 - 92.7	100	85.7 - 100	4.5	100	100	99.8
TM6SF1	0.807	0.674 - 0.941	< 0.0001	70	52.1 - 83.3	100	85.7 - 100	40.5	100	100	99.7
ZNF671	0.858	0.7437- 0.979	< 0.0001	83	66.4 - 92.7	100	85.7 - 100	22.0	100	100	99.8
COL6A2	0.831	0.713 - 0.949	< 0.0001	67	48.8 - 80.8	100	85.7 - 100	26.0	100	100	99.7
APC	0.587	0.423 - 0.751	0.2816	60	42.3 - 75.4	83	62.9 - 93.0	0.5	0	97	17.6
HIST1H3C	0.566	0.409 - 0.723	0.4142	33	19.2 - 51.2	100	85.7 - 100	20.5	100	99	99.3

AUC, Area under the ROC curve; CI, Confidence Interval of AUC;Maximized for sensitivity while retaining specificity of at least 90%; Population prevalence approximated at 1%.
Training set samples: colon carcinoma (N = 30) and adjacent normall (N= 23)

TEST SET - Panel Marker Performance											
Panel	ROC AUC	AUC 95% CI	AUC P-value	Sensitivity %	Sens. 95% CI	Specificity %	Spec. 95% CI	ROC Threshold	PPV %	NPV %	Accuracy %
13 - gene	1.00	1.00 - 1.00	< 0.0001	100	89.6 - 100	94.7	75.4 - 99.7	88.6	100.0	100.0	100.0
6 - gene	1.00	1.00 - 1.00	< 0.0001	100	88.6 - 100	89.5	68.6 - 98.1	26.0	16.1	100.0	94.8

AUC, Area under the ROC curve; CI, Confidence Interval of AUC; Maximized for sensitivity while retaining specificity of at least 90%; Population prevalence approximated at 1%.
Test set samples: colon carcinoma (N = 33) and adjacent normall (N= 19)

Table S1. ROC AUC, sensitivity, specificity, PPV, NPV and accuracy analyses for 13 individual methylated genes in fresh frozen colon carcinoma and normal colon tissue. Receiver operating characteristics (ROC) analyses was performed on QM-MSP data of training and test set samples. In the training set samples, 11 of 13 individual markers distinguished colon carcinoma from normal tissues (adjacent to tumor; excluding *APC* and *HIST1H3C)* as shown by moderate to high AUC (area under the curve; $P < 0.0001$ to 0.0007). Data analyses of the test set for 13- and a 6-marker subset showed that cumulatively the 13 -marker panel achieved high ROC AUC of 1.00, sensitivity of 100%, specificity of 94.7%, Positive predictive value (PPV) and negative predictive value (NPV) were 100%, with 100% accuracy. The 6-marker panel had a lower specificity of 89.5%, resulting in a PPV of 16.1%, and NPV of 100% with 94.8% accuracy, possibly a reflection of the small sample sets analyzed. These results support data in Table 2, Figs. 3-4, and Additional file 1 Fig. S1.
